# Broadly neutralizing monoclonal antibodies against human adenovirus types 55, 14p, 7, and 11 generated with recombinant type 11 fiber knob

**DOI:** 10.1038/s41426-018-0197-8

**Published:** 2018-12-08

**Authors:** Xingui Tian, Ye Fan, Zhenwei Liu, Ling Zhang, Jiayi Liao, Zhichao Zhou, Xiao Li, Tiantian Liu, Wenkuan Liu, Hongling Qiu, Rong Zhou

**Affiliations:** 1 State Key Laboratory of Respiratory Disease, National Clinical Research Center for Respiratory Disease, Guangzhou Institute of Respiratory Health, The First Affiliated Hospital of Guangzhou Medical University, Guangzhou Medical University, Guangzhou, China; 2 Department of Medical Genetics, Liuzhou Maternal and Child Health Hospital, Liuzhou, Guangxi China; 3 Shanghai Institute of Nutrition and Health, Chinese Academy of Sciences, Shanghai, China

## Abstract

The re-emerging human adenovirus types HAdV7, HAdV14, and HAdV55 of species B have caused severe lower respiratory tract diseases and even deaths during recent outbreaks. However, no adenovirus vaccine or therapeutic has been approved for general use. These adenoviruses attach to host cells via the knob domain of the fiber, using human desmoglein 2 as the primary cellular receptor. In this study, a recombinant HAdV11 fiber knob trimer (HAdV11FK) expressed in *E. coli* was shown to induce broadly neutralizing antibodies against HAdV11, -7, -14p1, and -55 in mice. Using HAdV11FK as an antigen, three monoclonal antibodies, 6A7, 3F11, and 3D8, with high neutralizing activity were generated. More importantly, the results of in vitro neutralization assays demonstrated that 3F11 and 3D8 cross-neutralized HAdV11, -7, and -55, but not HAdV14p1. The amino acids 251KE252 within the F-G loop may be the crucial amino acids in the conformational epitope recognized by 3F11, which is common to HAdV11, -7, -14p, and -55, but is not present in HAdV14p1 and HAdV3. A two-amino-acid deletion in the HAdV14p1 structure breaks the short alpha helix (248SREKE252) that is present in the HAdV7, -11, -55, and -14p fiber knob structures. Our findings add to the knowledge of adenovirus fiber structure and antibody responses and are important for the design of adenovirus vaccines and antiviral drugs with broad activity.

## Introduction

Human mastadenoviruses (HAdVs) are non-enveloped, double-stranded DNA viruses belonging to the family Adenoviridae. To date, more than 85 HAdV types, which are classified within seven species (A–G), have been identified and defined using a new paradigm based on genomics^1–4^. HAdVs are highly contagious pathogens that commonly cause respiratory diseases, including the common cold, tonsillitis, bronchitis, and severe pneumonia, and they can also bring about other diseases, such as gastroenteritis, cystitis, conjunctivitis, carditis, and meningoencephalitis, depending on the infection type. HAdV infections can occur in patients of all ages and susceptible populations, include infants, school students, military recruits, and immunocompromised patients^5,6^.

HAdVs of species B can be divided into two subspecies, B1, which includes HAdV3, -7, -16, -21, -50, and B2, which includes HAdV11, -14, -34, -35, and -55^7^. Unlike other HAdVs, HAdV3, -7, -11, -14, and -55 use human desmoglein 2 (DSG2) as the primary high-affinity receptor^8^. Of these HAdVs, HAdV3, -7, -14, and -55 have been reported to cause severe community-acquired pneumonia outbreaks in military and civilian populations^9^. HAdV3 and -7 are the most commonly detected in pediatric patients with respiratory infections, and HAdV7 is more likely to cause severe pneumonia and death than HAdV3^10–13^. HAdV7 is also one of the most commonly detected types associated with febrile acute respiratory disease (ARD) outbreaks in the military^14^. HAdV14 (also known as “agent de Wit” or HAdV14p) was first discovered in outbreaks of ARD in 1955 and then vanished for a long period of time. In 2006, a new HAdV14 strain designated “HAdV14p1” re-emerged in the USA among both civilian and military populations, causing at least 10 deaths^15^. Outbreaks of HAdV14p1 infections were subsequently reported in Europe^16^, Canada^17^, and Asia^18^. HAdV55 re-emerged in 2005 in Singapore, where it was first identified as HAdV11a. After the first reported outbreak in China in 2006, HAdV55 has caused numerous outbreaks in China among military recruits and civilians, and has become a common pathogen causing life-threatening pneumonia^13,19–22^. Our recent survey on seroprevalence indicates that there is a lack of herd immunity to HAdV14 and HAdV55 in civilian populations^23,24^. The high morbidity and mortality in otherwise healthy, immunocompetent adults render HAdV7, -14, and -55 potential threats to public health. Adenovirus infections in immunocompromised patients tend to become disseminated and severe and are associated with case fatality rates as high as 60% in patients with pneumonia and 50% in patients with hepatitis. Adenovirus infections are detected in 11% of transplant recipients, with case fatality rates from 60% for bone marrow transplant patients to 18% for renal transplant patients. HAdV-7 and -11 are the predominant types observed in bone marrow and renal transplant patients, respectively^25^, while HAdV11 is the most commonly detected type in patients with hemorrhagic cystitis^26^.

Currently, no vaccine for use in general populations and no efficient antiviral therapy for HAdVs is available. Neutralizing monoclonal antibodies (MAbs) are promising prophylactics or therapeutic drugs against viral diseases. The generation of neutralizing MAbs is useful in identifying neutralizing epitopes, an important step in the design of novel vaccines, antiviral drugs, and rapid diagnostic reagents.

The HAdV capsid is composed of three major proteins, the hexon, penton base, and fiber. The penton base forms the twelve vertices of the icosahedral capsid and stimulates clathrin-mediated endocytosis; the fiber protein protrudes from each vertex and mediates specific, high-affinity binding to the primary cellular receptors (e.g., CAR, CD46, and hDSG2); and the hexon protein is the most abundant capsid protein. Previous studies have confirmed that the hexon protein is the predominant target of neutralizing antibodies (NAbs) against HAdV3, -5, -7, -14, or 55^23,27–31^^.^ The type-specific neutralization epitopes on hexon proteins of many adenoviruses have been shown to be located primarily in seven hypervariable regions^29,31–36^. The fiber protein is composed of three portions. a tail, a shaft, and a knob. A few studies have demonstrated that subdominant but still functionally relevant NAbs are directed against the fiber knob in sera from vaccinated mice and humans and from naturally exposed humans^27,29,30^. Recombinant fiber knob can specifically distinguish antibodies against different HAdV species^37^. Recently, Feng et al. reported that hexon elicits type-specific NAbs, whereas fiber induces cross-neutralizing Abs to HAdV14 and HAdV55^31^. Previous studies have mapped linear epitopes on the fiber knobs of Ad2, HAdV3, HAdV5, Ad8, and Ad15 with synthetic peptides^38–41^. Lang et al. reported that the dominant neutralization epitopes were located primarily in the N-terminal region of knobs from Ad1, Ad2, and HAdV5, but they appeared to be located in the C-terminal region of the Ad6 knob, with some individual differences observed in rabbit and human populations^42^. However, only a few neutralization epitopes on HAdV fiber knob have been identified.

The most notable genetic difference between the variant HAdV14p1 and the prototype HAdV14p is a deletion of 6 base pairs in the fiber knob gene^15^. HAdV55 is an intertype recombinant of HAdV11 and -14 that contains the major hexon gene from HAdV11 and additional parts from HAdV14^22,43–45^. An amino acid sequence alignment indicates that HAdV55 shares the fiber knob with HAdV14p but not HAdV14p1. In addition, the fiber proteins of HAdV7, -11, -14p1, and -55 show more than 90% amino acid sequence similarity.

In this study, we expressed the HAdV11 fiber knob trimer in *E. coli* with the goal of generating an antigen that induces broadly neutralizing antibodies against HAdV11, -7, -14, and -55, resulting in the identification of three neutralizing MAbs. Subsequently, cross-reactions of the MAbs against HAdV11, -7, -14, and -55 were evaluated, and the shared crucial fiber knob amino acid residues and the conformational neutralization epitope were identified.

## Results

### Recombinant HAdV11 fiber knob induces cross-neutralizing antibodies against several types of HAdVs of species B

**Fig. 1:**
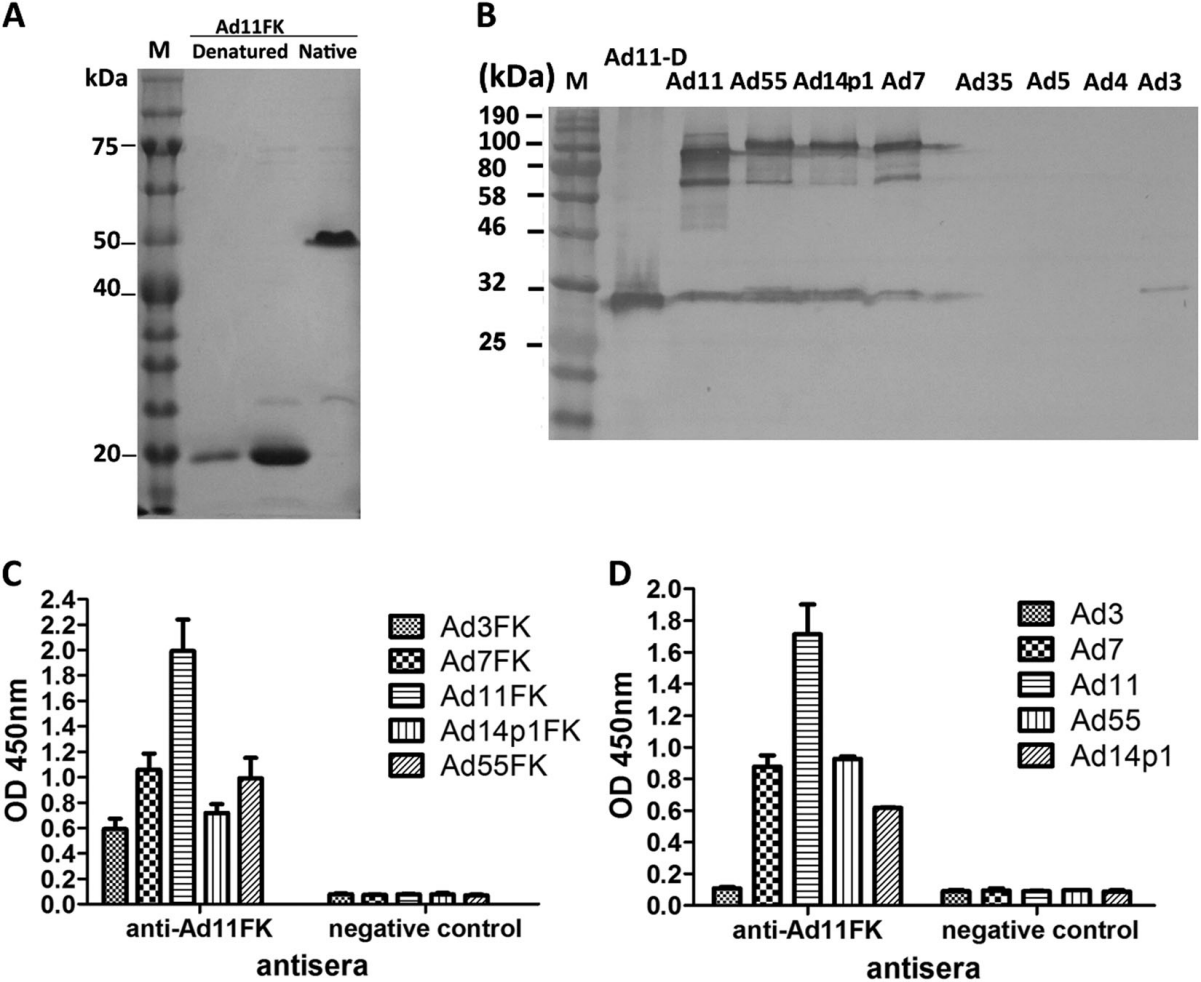
Recombinant HAdV11 fiber knob induces cross-reactive antibodies against several types of HAdV-B. **a** SDS-PAGE of the purified recombinant HAdV11 fiber knob (HAdV11FK). Purified HAdV11FK was mixed with 5 × loading buffer, incubated at room temperature for 5 min, and then incubated on ice (native) or heated at 98 °C for 5 min (denatured). **b** Immunoblot analysis indicated that the anti-HAdV11FK serum recognizes the HAdV11 fiber in its trimeric form and cross-reacts fibers of HAdV7, HAdV14p1, and HAdV55. Purified HAdV3, HAdV4, HAdV5, HAdV7, HAdV11, HAdV14p1, HAdV35, and HAdV55 virions were stored at room temperature or heated at 98 °C (HAdV11-D) for 5 min in the presence of loading buffer. The membranes were subsequently incubated with antiserum from a mouse immunized with recombinant HAdV11FK. M, PM5000 ExcelBand™ 3-color Pre-+Stained Protein Ladder, Regular Range (SMOBIO, Taiwan (R.O.C.)). **c**, **d** Cross-reactions of anti-HAdV11FK sera with recombinant HAdV3, HAdV7, HAdV11, HAdV14p1 and HAdV55 fiber knobs (**c**) or purified HAdV3, HAdV7, HAdV11, HAdV14p1 and HAdV55 virions (**d**) were detected by ELISA. Antiserum from mice immunized with PBS was used as the negative control. Each experiment was repeated independently at least three times, and the means ± standard deviations are shown. OD450nm, optical density at 450 nm

**Fig. 2:**
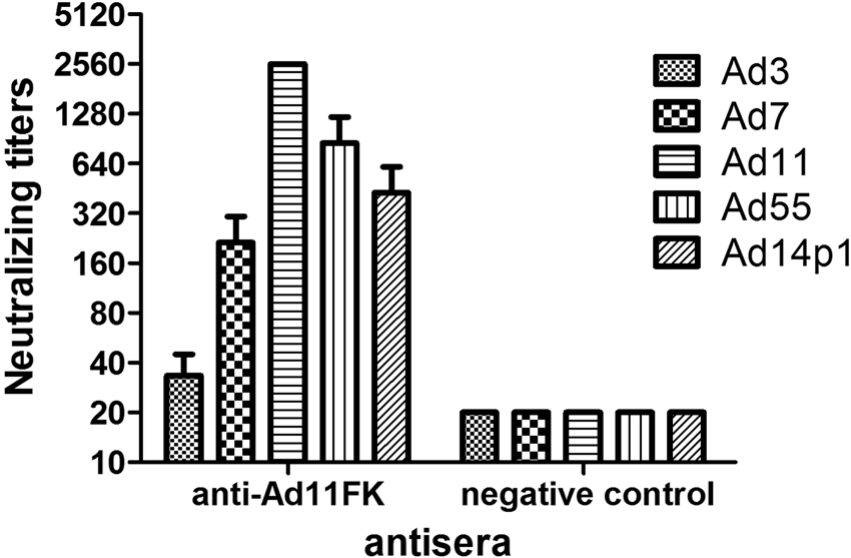
Cross-neutralizing antibody responses induced by HAdV11FK in mice. Sera from HAdV11FK-immunized mice (*n* = 3 per group) were assessed for NAb titers to HAdV3, HAdV7, HAdV11, HAdV55, or HAdV14p1 viruses. Antiserum from a mouse immunized with PBS was used as the negative control

A recombinant HAdV11 fiber knob peptide with the last repeat in the shaft (amino acids 234–425) of the fiber and an N-terminal His tag, HAdV11FK, was successfully expressed and purified as a soluble trimer (Fig. 1a). The HAdV11FK peptides maintained their trimeric form in SDS-PAGE loading buffer containing 2% SDS at room temperature and migrated as trimers in SDS-PAGE (native), whereas the peptides were detected in a monomeric form when heated at 98 °C for 5 min (denatured). Immunoblot analysis indicated that mouse anti-HAdV11FK serum recognizes the HAdV11 fiber in its trimeric form and cross-reacts with trimeric fiber from HAdV7, HAdV14p1, and HAdV55 but not HAdV3, HAdV4, HAdV5, and HAdV35 (HAdV11-D) (Fig. 1b). The HAdV11 fiber was also detected in its monomeric, denatured form using the anti-HAdV11FK serum (Fig. 1b). Interestingly, the anti-HAdV11FK serum cross-reacted with the recombinant fiber knob peptides of HAdV3, HAdV7, HAdV14p1, and HAdV55 (Fig. 1c), and with HAdV7, HAdV14p1, and HAdV55 virions but not HAdV3 virions (Fig. 1d) as indicated by ELISA. More importantly, the anti-HAdV11FK serum neutralized HAdV11 and cross-neutralized HAdV7, HAdV14p1, and HAdV55, while exhibiting a weak neutralization activity against HAdV3 (Fig. 2).

### Preparation of neutralizing MAbs directed against HAdV11 fiber knob

Of the 14 anti-HAdV11FK MAbs generated, three MAbs, 6A7, 3F11, and 3D8, had high neutralizing activity against HAdV11 in AD293 cells (Table 1). All three neutralizing MAbs were of the IgG1/κ isotype. The ascites titers of the three MAbs were determined by ELISA against HAdV11FK and were approximately 4,000,000 to 8,000,000. The neutralization titers of the MAbs 6A7, 3F11, and 3D8 were calculated to be 0.44, 0.66, and 1.05 μg/ml, respectively.

**Table 1:** Generation of Ad11-neutralizing MAbs

MAb	Isotype	Ascites titer^a^	IgG concentration (mg/ml)^b^	Neutralization titer against Ad11^c^
6A7	IgG1/κ	8,000,000	5.65	2560
3F11	IgG1/κ	4,000,000	4.24	1280
3D8	IgG1/κ	4,000,000	6.72	1280

^a^Ascites titer against Ad11FK is expressed as the reciprocal of the ascites dilution and was determined by indirect ELISA

^b^Each ascitic fluid sample (1 ml) was purified by octanoic acid–ammonium sulfate precipitation to a final volume of 200 μl and the IgG concentration was determined spectrophotometrically using a conversion factor of 1.4 mg/ml per absorbance unit at 260 nm

^c^Neutralization titer is expressed as the reciprocal of the ascites dilution and was determined as the highest dilution of ascites that protected AD293 cell monolayers from an obviously observable CPE

### Cross-neutralization of the MAbs against other types of HAdVs of species B

**Fig. 3:**
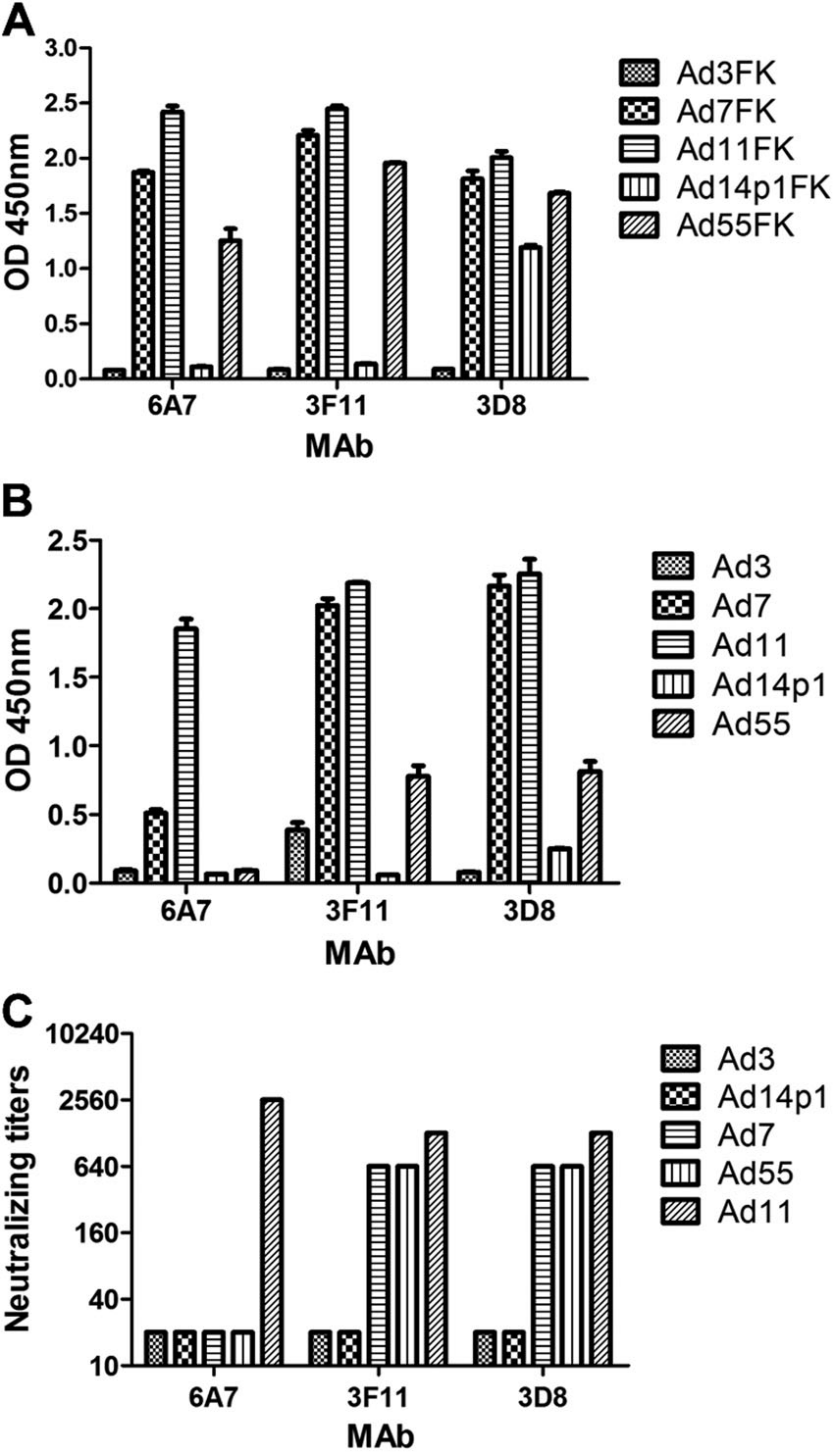
MAbs cross-react with various HAdV types. The reactions of MAbs with recombinant HAdV3, HAdV7, HAdV11, HAdV14p1, and HAdV55 fiber knobs (**a**) or purified HAdV3, HAdV7, HAdV11, HAdV14p1, and HAdV55 virions (**b**) were detected by ELISA. Each experiment was repeated independently at least three times, and the means ± standard deviations are shown. OD450nm, optical density at 450 nm. **c** In vitro micro-neutralization test of MAbs. The MAbs were assessed for NAb titers to HAdV3, HAdV7, HAdV11, HAdV55, or HAdV14p1 viruses

The anti-HAdV11FK serum cross-reacted with HAdV11, HAdV7, HAdV14p1, HAdV55, and HAdV3, demonstrating that it is possible to prepare MAbs that cross-react with several HAdV types. When the recombinant fiber knob peptides were used as a coating antigen for ELISA (Fig. 3a), the MAbs 6A7 and 3F11 reacted with HAdV11FK, HAdV7FK, and HAdV55FK but not with HAdV3FK and HAdV14p1FK, while the MAb 3D8 reacted with HAdV11FK, HAdV7FK, and HAdV55FK and weakly reacted with HAdV14p1FK. When purified virions were used as coating antigen for ELISA (Fig. 3b), the MAb 6A7 reacted with HAdV11 and weakly reacted with HAdV7 but did not react with HAdV3, HAdV14p1, or HAdV55; the MAb 3F11 reacted with HAdV11, HAdV7 and HAdV55 but not with HAdV3 and HAdV14p1; and the MAb 3D8 reacted with HAdV11, HAdV7, and HAdV55 and weakly but detectably reacted with HAdV14p1.

Importantly, in vitro neutralization assays demonstrated that the MAbs 3F11 and 3D8 cross-neutralized HAdV11, HAdV7, and HAdV55 but not HAdV14p1, whereas the MAb 6A7 did not neutralize HAdV7, HAdV55, or HAdV14p1 (Fig. 3c).

**Fig. 4:**
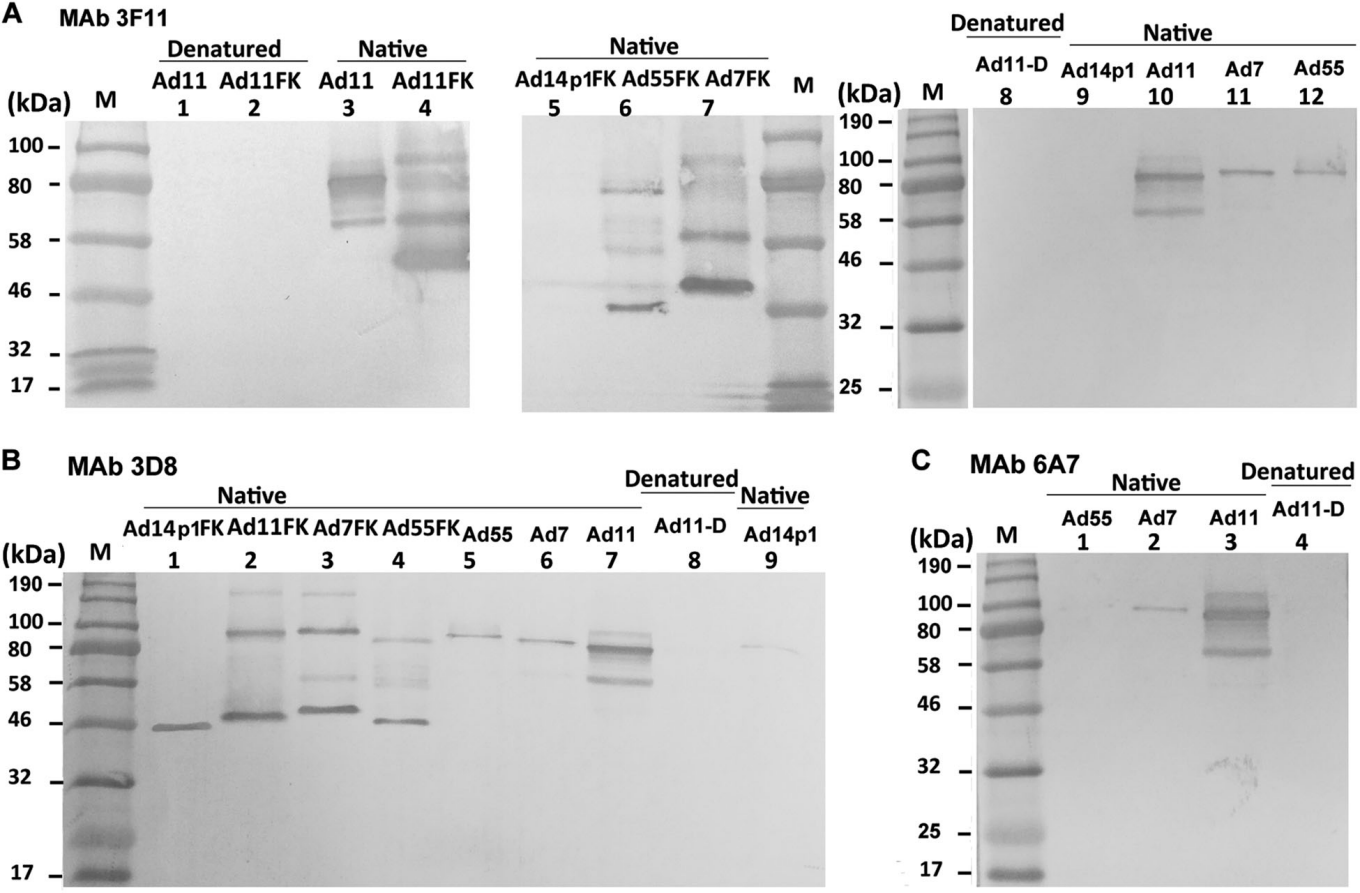
Immunoblot analysis indicates MAbs recognize the HAdV11 fiber in its polymeric form. Purified HAdV7, HAdV11, HAdV14p1, and HAdV55 virions or recombinant fiber knob (FK) were stored at room temperature (“native”) or heated at 98 °C (“denatured”) for 5 min in the presence of loading buffer. The membranes were subsequently incubated with the MAbs 3F11 (**a**), 3D8 (**b**) or 6A7 (**c**), after which they were incubated with an HRP-conjugated secondary antibody and developed with TMB substrate. M, standard prestained protein marker (NEB, Hitchin, UK)

Native and denatured western blot analyses were performed to determine whether the MAbs recognized the antigens in a conformation-dependent manner (Fig. 4). At room temperature, fiber or fiber knob maintained its trimeric form in SDS buffer. The theoretical molecular weight of each of the HAdV7, HAdV11, HAdV14p1, and HAdV55 fiber monomers is approximately 35 kDa, while that of the HAdV7, HAdV11, HAdV14p1, and HAdV55 fiber knob monomers is approximately 22 kDa. The MAb 3F11 only recognized the native trimeric HAdV11 fiber (lanes 3 and 10) and fiber knob (HAdV11FK) (lane 4) but not monomeric HAdV11 fiber (lanes 1 and 8) or fiber knob (HAdV11FK) (lane 2) generated by denaturation at 98 °C in the presence of SDS (Fig. 4a). The MAb 3F11 also recognized the native trimeric HAdV7 fiber (lane 11) and fiber knob (lane 7) and that of the HAdV55 fiber (lane 12) and fiber knob (lane 6) but not that of the HAdV14p1 fiber (lane 9) and fiber knob (lane 5) (Fig. 4a). The MAb 3D8 recognized the native trimeric HAdV11 fiber (lane 7) and fiber knob (lane 2) but not the denatured monomeric HAdV11 fiber (lane 8) (Fig. 4b). The MAb 3D8 also recognized the native trimeric HAdV7 fiber (lane 6) and fiber knob (lane 3) and that of the HAdV55 fiber (lane 5) and fiber knob (lane 4) (Fig. 4b). The MAb 3D8 reacted with trimeric HAdV14p1 fiber knob (lane 1), while the MAb 3D8 weakly but detectably reacted with trimeric HAdV14p1 fiber (lane 9), which is essentially in agreement with the ELISA results. Interestingly, the MAb 3D8 reacted with the hexameric HAdV11 (lane 2), HAdV7 (lane 3), and HAdV55 fiber knobs (lane 4) but not the hexameric HAdV14p1 fiber knob (lane 1) (Fig. 4b). The MAb 6A7 only recognized the native trimeric HAdV11 fiber (lane 3) but not the denatured monomeric HAdV11 fiber (lane 4) (Fig. 4c). The MAb 6A7 also recognized the native trimeric HAdV7 fiber (lane 2) but not the HAdV55 fiber (lane 1) (Fig. 4c). These results suggested that all three antibodies recognize epitopes on the fiber homotrimer of HAdV11 in a conformation-dependent manner.

### Epitope identification by sequence alignment and antigen-antibody molecular docking analyses

**Fig. 5:**
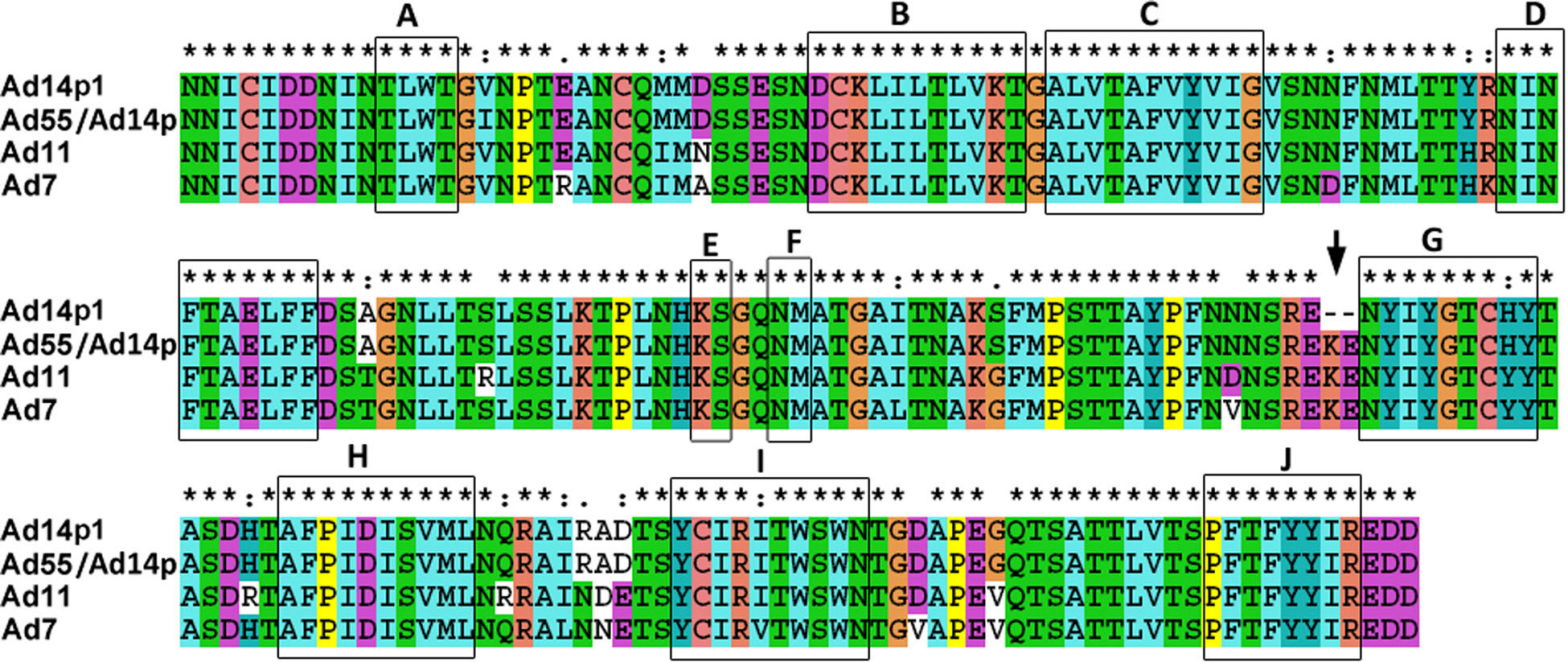
Amino-acid sequence alignment of the fiber knobs from several HAdV species B. “*”, conserved amino acid; “.”, either size or hydropathy is conserved; and “:”, both size and hydropathy are conserved. Gaps used to optimize alignments are indicated by dashes. Beta sheets **a**–**j** present in the knobs are indicated by rectangles. The deletion of two amino acid residues (251KE252) within the F-G loop of the HAdV14p1 fiber knob is indicated by an arrow

The most notable genetic difference between the variant HAdV14p1 and the prototype strain HAdV14p is a 6-base pair deletion in the fiber knob gene. HAdV55 evolved from an intertypic recombinant event in the hexon gene between the renal pathogen HAdV11 and the respiratory pathogen HAdV14p, with HAdV55 containing the major hexon gene from HAdV11 and additional parts, including the fiber gene, from HAdV14p. HAdV55 and HAdV14p have the same fiber knob amino acid sequence (not shown), and an alignment of the HAdV11, HAdV7, HAdV14p1, and HAdV55 fiber knob peptide sequences revealed variations in a few amino acids (Fig. 5). The alignment between the HAdV14p1 and HAdV55 fiber proteins revealed only three amino acid variations in the fiber knob region, an amino acid substitution of 138I (HAdV55 fiber) vs. 138 V (HAdV14p1 fiber), and a deletion of two amino acids in HAdV14p1 fiber (249R--E250) vs. HAdV55 fiber (249REKE252). The corresponding amino acids of the HAdV7 and HAdV11 fibers are 138 V, identical to that of HAdV14p1, and 249REKE252, which is present in HAdV55. The MAbs 3F11 and 3D8 neutralized HAdV11, HAdV7, and HAdV55 but not HAdV14p1 (Fig. 3c). The MAb 3F11 recognized the HAdV11, HAdV7, and HAdV55 fiber knobs but not that of HAdV14p1 by ELISA or by western blot analyses (Figs. 3 and 4). These results suggested that the amino acids 251KE252 may be crucial in the conformational epitope recognized by the neutralizing MAb 3F11. Thus, the epitope is shared by HAdV11, HAdV7, HAdV14p, and HAdV55 but not HAdV14p1.

**Fig. 6:**
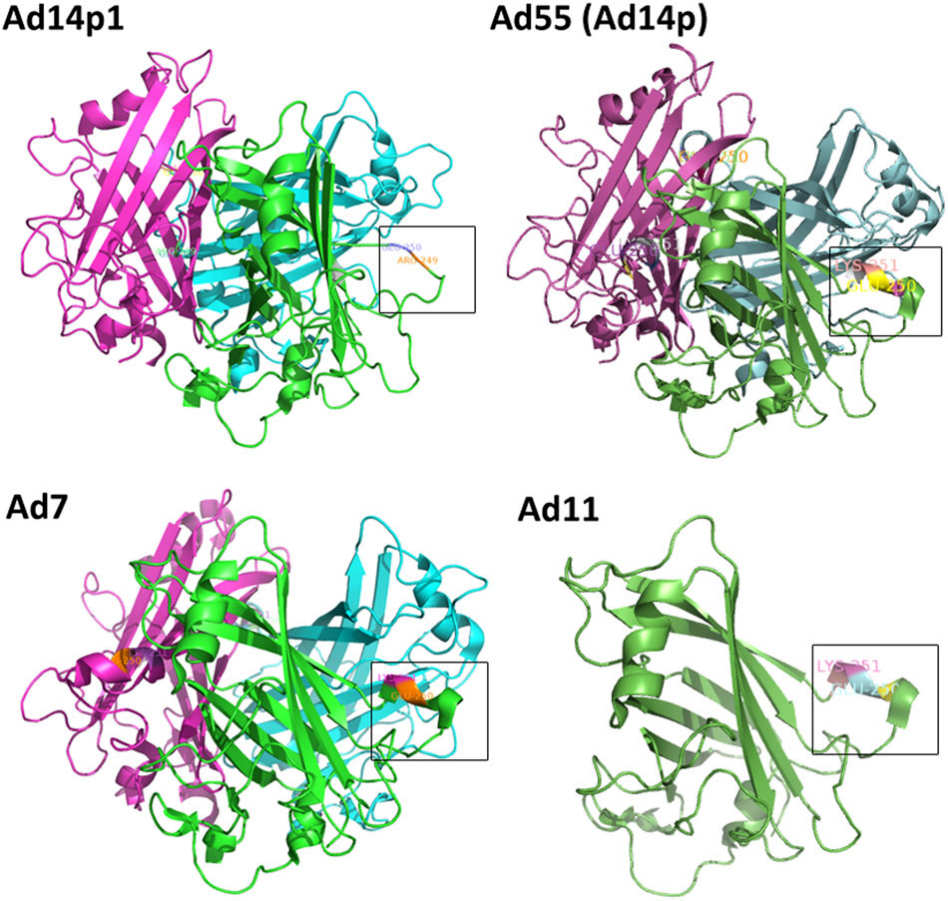
Structural models of homotrimeric HAdV7, -14p1, and -55 (-14p) fiber knobs and monomeric HAdV11 fiber knob. PyMOL v0.99 was used to generate the cartoon representation of the fiber knob structures. The partial residues for the F-G loop are shown in frame and are numbered according to the fiber. The EK deletion in HAdV14p1 breaks the alpha helix, as indicated

**Fig. 7:**
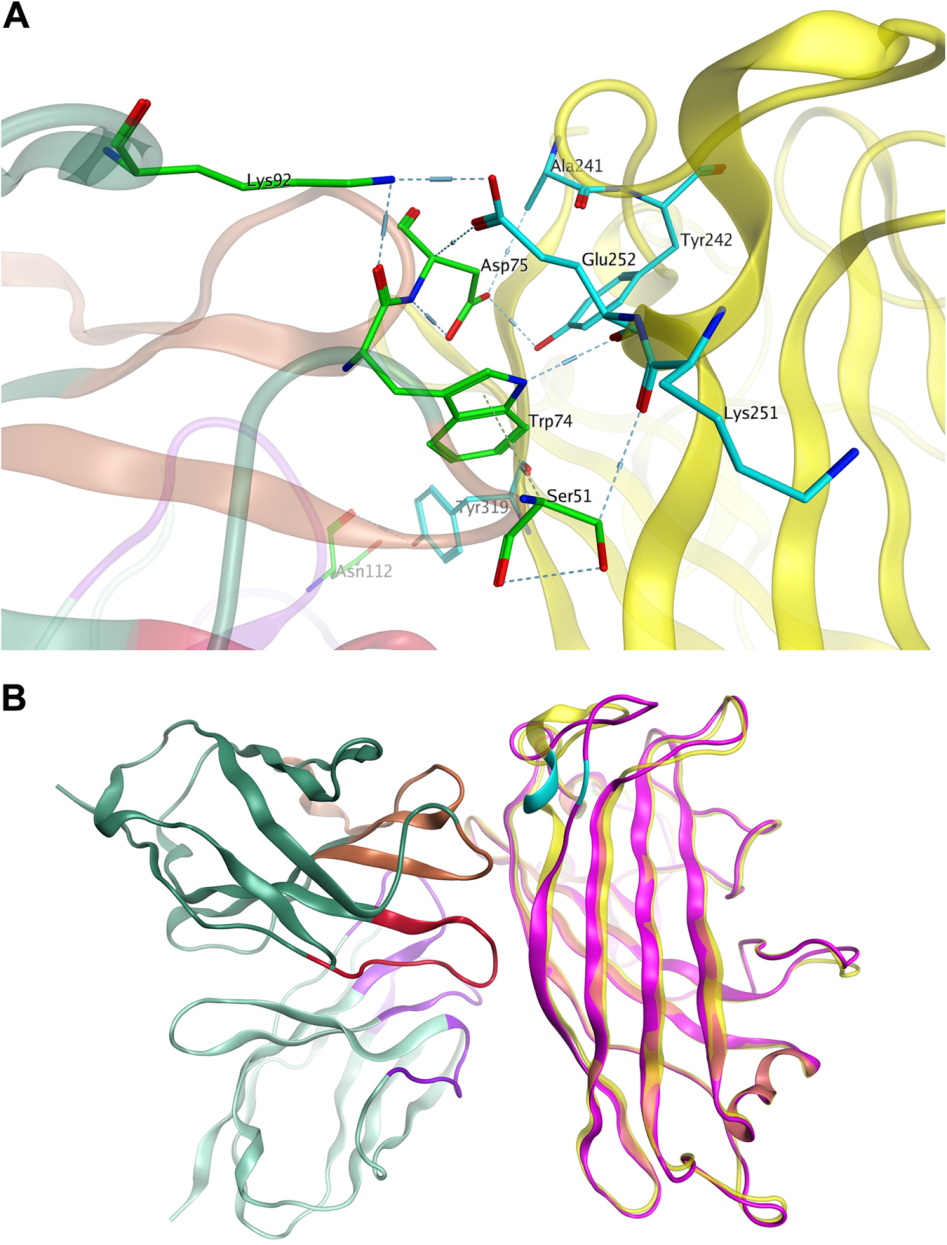
Molecular docking of MAb 3F11 to the HAdV11FK antigen. **a** Major binding interface between the MAb 3F11 and HAdV11FK. The residues involved in strong intermolecular contacts are shown in stick representations. The antibody residues are colored in green, and the antigen residues are colored in cyan. Strong polar interactions, such as hydrogen bonds and salt bridges, are shown in light blue dashes. **b** Overview of the docked complex of the antigen HAdV11FK and the antibody Fv, superimposed with HAdV14p1FK. The protein backbones are shown in ribbons. The antigen HAdV11 is colored in yellow, and HAdV14p1FK is colored in magenta. The antibody Fv is colored in dark green (FR), purple (CDR-L1/L2/L3), brown (CDR-H1/H2), and red (CDR-H3), respectively. The location of E252 on the antigen is colored in cyan

The deletion (251KE252) in the fiber knob is located within the F-G loop directly adjacent to the G β-sheet and could theoretically change the structure of the F-G loop and its proximity to the neighboring knob monomer (Fig. 5)^46,47^. The loop exhibits a highly flexible secondary structure. As shown in Fig. 6, the two-amino-acid deletion in the HAdV14p1 structure breaks the short alpha helix (248SREKE252) that is present in the HAdV7, -11, HAdV55, and -14p structures. Molecular docking of the MAb 3F11 to HAdV11FK suggested that R249, K251, and E252 of HAdV11FK may strongly interact with the MAb 3F11 CDR H1/H2 by polar interactions, such as hydrogen bonds and salt bridges (Fig. 7a). The skeleton conformation changed from the helix of HAdV11FK to the loop of HAdV14p1FK, which may be the primary cause of the failure to bind to the antibody (Fig. 7b).

## Discussion

In this study, we report that recombinant trimeric HAdV11 fiber knob induced cross-neutralizing antibody responses against HAdV11, -7, -14p1, and -55 in mice. Using HAdV11FK as an antigen, three neutralizing MAbs, 6A7, 3F11 and 3D8, were obtained, of which 3F11 and 3D8 cross-neutralized HAdV11, -7, and -55 but not HAdV14p1. Furthermore, two crucial amino-acid residues (251KE252) and a conformational epitope that are common to the HAdV11, -7, -14p, and -55 fiber knobs, which are deleted in HAdV14p1, are recognized by the MAb 3F11.

Surprisingly, antiserum against HAdV11FK neutralized other HAdV types, including HAdV7, -14, and -55. Thus, recombinant HAdV11FK may be a vaccine candidate against these HAdVs, which should be evaluated in future work. It is generally believed that HAdV NAbs are serotype-specific, and some previous studies have demonstrated that they show minimal or no cross-reactivity to different HAdV species^37,48,49^. However, few studies have reported on the cross-neutralization of different types of the same HAdV species. Recently, Feng et al. demonstrated that a fiber-specific antibody in sera contributed to cross-neutralizing activity against HAdV14 and HAdV55^31^. However, HAdV55 is known to be an intertype recombinant of HAdV11 and -14 that contains the fiber gene from HAdV14. Phylogenetic analysis of species B fiber proteins revealed that HAdV7, -11, -14, and -55 belong to the same branch, and among the fibers or fiber knobs of these HAdVs exhibit similarities of more than 90%. However, the similarities among fibers of different branches were <63% (not shown). The high similarity may explain the broadly neutralizing activity of the anti-HAdV11FK serum against HAdV7, -14, and -55 (Figs. 1 and 2). We observed that the similarities among the fiber proteins of HAdV21, -34, -35, and -50 were also greater than 90%. Whether the fibers of these HAdV types induce cross-neutralizing antibodies requires further study.

The recombinant adenovirus fiber knobs were expressed and purified as soluble trimers in *E. coli*. In SDS-PAGE loading buffer containing 2% SDS and without heating, the HAdV11FK peptide and HAdV11 fiber protein maintained their trimeric forms. The non-linear structure may explain why the observed molecular weight (~54 kDa) of the HAdV11FK trimer was lower than the theoretical molecular weight (68 kDa). The anti-HAdV11FK serum recognized the HAdV11 fiber in its trimeric form but did so weakly in its monomeric form. This result suggests that natural trimeric HAdV11FK induces antibodies that are directed primarily at conformational epitopes. All three monoclonal antibodies with neutralizing activity exclusively recognized, the trimeric form of the fiber, indicating that this form is necessary for inducing neutralizing antibodies and making contact with the receptor(s).

Using HAdV11FK as an antigen, three neutralizing MAbs, 6A7, 3D8, 3F11, were obtained. The MAbs 3F11 and 3D8 could neutralize HAdV11, HAdV7, and HAdV55 but not HAdV14p1. The fiber of HAdV55 is the same as that of HAdV14p. While we did not perform neutralization tests with MAbs against HAdV14p, we predict that the MAb 3F11 can neutralize HAdV14p. HAdV14 is an important focus of research with great clinical significance because of the recent appearance of a new, more pathogenic strain (HAdV14p1). The genomes of HAdV14p and HAdV14p1 differ by an insertion in the gene encoding E1A and a small deletion in the fiber knob-encoding gene. Some studies have delineated potential mechanisms for the higher pathogenicity of HAdV14p1 relative to HAdV14p. Wang et al. suggested that the altered three-dimensional structure of the HAdV14P1 fiber knob in the F-G loop region caused by the deletion (251KE252) does not significantly change the affinity of the fiber knob for hDSG2 or the intracellular signaling and DSG2 shedding of the virus in epithelial cancer cells^47^. The insertion in E1A may contribute to the higher pathogenicity of HAdV14p1. The authors also tested 78 human serum samples for IgG antibodies specific to recombinant HAdV14p and HAdV14p1 fiber knobs and observed that the antibody levels were significantly different in the majority of cases^47^. Thus, the two amino acids play an important role in immunoreactivity.

The MAb 3D8 cross-neutralized HAdV11, HAdV7, HAdV55 but not HAdV14p1. Thus, 251KE252 is crucial for the neutralization of the MAb 3D8. However, the MAb 3D8 can bind to HAdV14p1 trimeric but not hexameric fiber knob (Figs. 3a and 4b). Therefore, MAb 3D8 may recognize a common conformational epitope in hexameric fiber knobs of HAdV11, HAdV7, and HAdV55, which is also present in trimeric fibers of HAdV11, HAdV7, and HAdV55 viruses. 251KE252 is crucial for this conformational epitope, and some other amino acids may also be important for this epitope. The MAb 3D8 may also bind amino acids other than 251KE252 that form a conformational epitope in recombinant trimeric fiber knob of HAdV14p1. The structural conformation of recombinant trimeric fiber knob may be somewhat different from that of trimeric fiber. The MAb 6A7 was also observed to bind to the HAdV55 fiber knob but not the HAdV55 fiber (Fig. 3a, b). Wang et al. observed that multimerization of the HAdV3 trimeric fiber knob domain is required for efficient binding of the virus to the receptor desmoglein 2 and subsequent opening of epithelial junctions^50^. Our results indicate that the structural difference between the trimeric fiber knob and natural trimeric fiber may contribute to the incomplete inhibition of HAdV3 binding and infection. In this study, computer-generated molecular docking was performed to generate an interaction model of the MAb 3F11 binding to the HAdV11 fiber knob, which validated the importance of the residues 251KE252. However, the actual interaction during the binding of the MAbs 3F11 and 3D8 to fibers should be investigated by cryo-EM in future.

Our results indicated that the amino acids 251KE252 are crucial for a common and conformational neutralizing epitope of the HAdV11, -7, -55, and -14p fiber knobs. The preparation of broadly neutralizing MAbs further confirms the presence of a common neutralizing epitope in HAdVs of species B. Anti-HAdV11FK serum could neutralize HAdV11, -7, -55 as well as HAdV14p1, suggesting that there may be other common neutralizing epitopes for the HAdV11 and -14p1 fiber knobs. This is the first report of MAbs broadly neutralizing several different HAdV types. In future work, it should be investigated whether the MAbs can provide protection against these HAdVs in vivo, after which the MAbs may be further humanized for use as therapeutics.

In summary, we generated recombinant HAdV11FK that induced high-titer cross-neutralizing antibodies against HAdV11, -7, -14p, and -55. We prepared three MAbs using HAdV11FK as an antigen, of which 3F11 and 3D8 were shown to be broadly neutralizing MAbs against HAdV11, -7, -14p, and -55. We also identified a conformational epitope common to the HAdV11, -7, -55, and -14p fiber knobs using the MAb 3F11. These results add to the knowledge of HAdV fiber structure and antibody responses and provide insights into the design of HAdV vaccines and therapeutics.

## Materials and methods

### Viral strains and cells

The HAdV11 Slobitski strain (GenBank accession no. AF532578.1) and HAdV35 Holden strain (GenBank accession no. AY128640.2) from ATCC, the HAdV3 GZ01 strain (GenBank accession no. DQ099432)^51^, the HAdV-4 strain GZ01 (GenBank accession no. KF006344.1)^36^, the HAdV7 GZ08 strain (GenBank accession no. GQ478341.1)^28^, the HAdV14p1 GZ01 strain (GenBank accession no. JQ824845.1)^52^, and the HAdV55 Shanxi-Y16 strain (GenBank accession no. KF911353.1)^53^ were maintained in our laboratory. All HAdVs were cultured in HEp-2 or AD293 cells, and HAdV particles were purified by standard CsCl gradient centrifugation as previously described^28^. Virus particle (VP) titers were determined spectrophotometrically using a conversion factor of 1.1 × 10^12^ VPs per absorbance unit at 260 nm^28^.

### Recombinant peptides and polyclonal antisera

A recombinant HAdV11 fiber knob peptide with the last shaft repeat (amino acids 123–325 of fiber) and an N-terminal His tag, HAdV11FK, was expressed from the vector pQE30 in *E. coli* and was purified by affinity chromatography using Ni-NTA His-Bind Resin (Novagen, EMD Millipore Corp., Billerica, MA, USA) under native conditions. The fiber knob peptides of HAdV3, HAdV4, HAdV7, HAdV14p1, HAdV55, and HAdV35 were also expressed in *E. coli* and were purified using the same method. The purified recombinant peptides were subsequently mixed with 5× loading buffer (10% sodium dodecyl sulfate [SDS], 5% 2-mercaptoethanol, 0.5% bromophenol blue, and 50% glycerol in 250 mM Tris-HCl [pH 6.8]), incubated at room temperature for 5 min, and then incubated on ice (native) or heated for 5 min at 98 °C (denatured). The samples were then separated by 12% SDS-polyacrylamide gel electrophoresis (PAGE).

Groups of five female BALB/c mice aged 4–6 weeks were immunized intraperitoneally with 40–50 μg/mouse of recombinant HAdV11FK emulsified with Freund’s complete adjuvant. Three booster doses were administered at 2-week intervals using the same dose of antigen and Freund’s incomplete adjuvant. Blood was collected from anesthetized mice via the retro-orbital lobe 10 days after the final immunization, and the sera were isolated, heat-inactivated, and stored frozen for subsequent serology tests.

The animal procedures used in this study were evaluated and approved by the Ethics Committee of the First Affiliated Hospital of Guangzhou Medical University (Guangzhou, China) and complied with all relevant guidelines and the National Law for Laboratory Animal Experimentation of China. The animal experiments were conducted in strict accordance with the recommendations of the Guide for the Care and Use of Laboratory Animals of the National Institutes of Health of the United States. All animals were housed individually and received humane care. During injection and sample collection, the mice were anesthetized with 1.5% isoflurane or 1 ml/kg body weight of 3% pentobarbital sodium to minimize their suffering.

### Generation of neutralizing monoclonal antibodies

Purified HAdV11FK was used to immunize BALB/c mice and to screen the resulting MAbs. The production and screening of mouse MAbs directed against HAdV11FK was carried out as described previously^54^. Briefly, BALB/c mice (6- to 8-weeks old) were injected intraperitoneally with 50 μg/mouse of recombinant HAdV11FK emulsified with Freund’s complete adjuvant and were boosted twice with the same dose of antigen and Freund’s incomplete adjuvant at 2-week intervals. Three days after intravenous boosting with 20 μg of HAdV11FK in phosphate-buffered saline (PBS) per mouse, the mice were killed and hybridoma fusion was performed using a standard protocol^55^. Hybridomas secreting HAdV11FK-specific antibodies were screened with an enzyme-linked immunosorbent assay (ELISA) using the HAdV11FK peptide. Positive hybridomas were subsequently subcloned by limited dilution. Ascites were generated by injecting the hybridoma cells into mice primed with Freund’s incomplete adjuvant. The ascites titers against HAdV11FK were determined by ELISA, and MAbs were purified from the ascites by octanoic acid–ammonium sulfate precipitation. MAbs were screened from the ascites with a virus neutralization assay. The IgG concentrations were determined spectrophotometrically using a factor of 1.4 mg/ml per absorbance unit at 260 nm. The antibody isotypes were determined using an ISOSTRIP Mouse Monoclonal Antibody Isotyping kit (Roche, Indianapolis, IN, USA), according to the manufacturer’s instructions.

### Indirect ELISA

For ELISAs, 96-well Nunc MaxiSorp™ flat-bottom plates (Nunc, Roskilde, Denmark) were coated with recombinant peptides (~2 μg/ml) or VPs (~10^10^ VPs/ml) in carbonate-buffered saline (pH 9.6) overnight at 4 °C. Next, the plates were washed once with 0.05% Tween-20 in PBS (PBST) and blocked for 2 h with 2% bovine serum albumin in PBST. MAb ascites (100 μl per well) or antiserum dilutions (from 1:100 to 1:1,000,000) was then added to each well and incubated at 37 °C for 1 h. The plates were washed three times with PBST and then incubated with a 1:10,000 dilution of horseradish peroxidase (HRP)-conjugated goat anti-mouse IgG (H + L) affinity-purified secondary antibody or HRP-conjugated goat anti-rabbit IgG (H + L) secondary antibody for 1 h. After the plates were washed five times with PBST, the products were visualized with tetramethylbenzidine (TMB) substrate. The reaction was stopped by the addition of 2 M H_2_SO_4_, and the results were analyzed with an ELISA plate reader (Multiskan MK3; Thermo Scientific) at 450 nm.

### Virus neutralization test

MAb ascites or antiserum pretreated at 56 °C for 30 min was serially diluted 2-fold with Dulbecco’s modified Eagle’s medium (Gibco, Beijing, China). Fifty-microliter aliquots of each dilution were mixed with an equal volume of wild-type adenovirus (50% tissue culture infective doses [TCID_50_]). The antibody virus mixtures were incubated at 37 °C for 1 h and then transferred to 96-well plates containing 85–95% confluent monolayers of AD293 cells. The monolayers were cultured for 48–72 h, after which titers from triplicate wells were read as the highest dilution of antiserum that notably inhibited the visible cytopathic effect (CPE).

### Immunoblotting analysis

AD293 cells were infected with wild-type adenovirus or the recombinant adenoviruses. At 72 or 96 h post-infection, the cells were harvested and lysed by freeze-thawed three times. The purified virion suspensions or purified recombinant peptides were then mixed with 5 × loading buffer (10% SDS, 5% 2-mercaptoethanol, 0.5% bromophenol blue, and 50% glycerol in 250 mM Tris-HCl [pH 6.8]), incubated at room temperature for 5 min, and then incubated on ice (native) or heated for 5 min at 98 °C (denatured). The samples were then separated by 12% SDS-PAGE and transferred electrophoretically onto polyvinylidene difluoride membranes. The membranes were blocked with 5% skim milk in PBS and then incubated with MAb ascites or mouse antiserum at a final dilution of 1:5,000. The membranes were washed again and then incubated with a 1:5,000 dilution of HRP-conjugated goat anti-mouse IgG secondary antibody or HRP-conjugated goat anti-rabbit IgG secondary antibody. After washing, the blots were developed with 1-Step Ultra TMB Blotting Solution substrate (Thermo Scientific, Rockford, IL, USA) at room temperature for 5 min.

### Bioinformatics analysis

The fiber knob crystal structures of HAdV11 (3exv.1.A), HAdV14p (3f0y.1.A), HAdV14p1 (4zdg.1.B), and HAdV7 (3exw.1.A) were downloaded from the Protein Data Bank. PyMOL v0.99 was used to generate cartoon representations of the fiber knob structures, and CLUSTALX was used for multiple sequence alignments of adenovirus proteins using the default parameters.

### Molecular docking of antibody to antigen

Total RNA was extracted from 100,000 hybridoma cells, and the antibody heavy and light chain variable region and joint region genes and alleles were amplified by RT-PCR for cloning and sequencing, with the sequences subsequently analyzed with IMGT/V-QUEST. Molecular Operating Environment (MOE) v2018.01 from Chemical Computing Group (Montreal, QC, Canada) was used to construct homology-based models of antibody structures. Amber10:EHT force field as implemented in MOE was used. The Fv region was built using the MOE antibody modeler by searching for appropriate template frameworks in the antibody database. The assigned templates for the FR regions were 1N5Y.L for VL and 1N5Y.H for VH. The assigned templates for the CDR loops were as follows: L1: 3WII.L, L2: 2A6D.L, L3: 1YNT.C, H1: 3RKD.H, H2: 3RKD.H, and H3: 3TT1.I. The maximum number of primary chain models and the number of side chain models per primary chain were both set to 25. Up to 625 intermediate models were built for further scoring. GB/VI solvation was used for ranking the intermediate models, and the model with the best GB/VI score was chosen for final energy minimization.

Antibody-antigen molecular docking was performed using ClusPro^56,57^. The antigen structure was preprocessed using MOE, including for hydrogenation, removal of crystalline water and small molecules, complementation of the missing residues (loop sampling and modeling when necessary), optimization of the protonation state, and for determining the necessary energy optimization. The ligand (antibody) was rotated using 70,000 rotations, with the ligand translated along the *x*, *y*, and *z* axes relative to the receptor on a grid for each rotation. One translation with the best score was chosen from each rotation. Of the 70,000 rotations, the 1000 rotations/translation combinations with the lowest scores were chosen. Next, a greedy clustering of these 1,000 ligand positions with a 9 Å C-alpha root mean square deviation radius was conducted to identify the ligand positions with the most “neighbors” in 9 Å, i.e., cluster centers. The ten cluster centers with the most cluster members were retrieved and individually inspected visually. The intermolecular contacts between the antibody and the antigen from the most probable pose were further evaluated.
